# Context, Development, and Digital Media: Implications for Very Young Adolescents in LMICs

**DOI:** 10.3389/fpsyg.2021.632713

**Published:** 2021-04-21

**Authors:** Lucía Magis-Weinberg, Ahna Ballonoff Suleiman, Ronald E. Dahl

**Affiliations:** ^1^Institute of Human Development, University of California, Berkeley, Berkeley, CA, United States; ^2^Department of Public Health, Sacramento State University, Sacramento, CA, United States; ^3^Department of Public Health, University of California, Berkeley, Berkeley, CA, United States

**Keywords:** very young adolescents, digital media, social media, LMICs, Global South

## Abstract

The rapidly expanding universe of information, media, and learning experiences available through digital technology is creating unique opportunities and vulnerabilities for children and adolescents. These issues are particularly salient during the developmental window at the transition from childhood into adolescence. This period of early adolescence is a time of formative social and emotional learning experiences that can shape identity development in both healthy and unhealthy ways. Increasingly, many of these foundational learning experiences are occurring in on-line digital environments. These expanding vulnerabilities and opportunities are being further amplified for young adolescents growing up in low resourced settings around the world. Cultural and contextual factors influence access, use, and appropriation of digital technology. Further, neurobehavioral changes associated with the onset of puberty often coincide with entry into social media and more autonomous use of technology. In low-and-middle-income countries (LMICs), disparities in access, use, and appropriation of digital media can amplify prevailing economic gaps, and compound gender inequalities during early adolescence. In LMICs, adolescents are often the early adopters of mobile technology and social media platforms. While the impact of social media on the well-being, particularly mental health, of young adolescents has been a focus of research in high-income countries (HICs), much less is known about the impacts of social media use on young adolescents in LMICs. In this paper, we review what is known about the interaction between digital media and early adolescent development. We highlight crucial gaps in the evidence in LMICs; and describe some hypotheses and areas for future research to address these compelling issues.

## The Promise of the Digital World

Youth are often the early adopters, rapid adapters, and innovators of new technologies—in ways that amplify risks and opportunities, impacting health, well-being, and trajectories of social and economic success. World-wide, social media has become one of the primary modes of digital media use—particularly for youth. The largest generation of adolescents in history is coming of age in the next decade. Most children below the age of 14 (89%) are growing up in low-and-middle-income countries (LMICs^1^;The World Bank, 2019)—and increasingly amidst rapid technological changes. However, most of the current literature exploring the relationship between digital media and well-being has been conducted with older adolescents growing up in high-income countries (HICs; Livingstone et al., 2017a; Schønning et al., 2020). Shifts in cultural and social context, coupled with the dynamic biological changes of early adolescence, inform how adolescents use digital media. A global developmental science lens can expand our understanding of the relationship between context, development, and digital media use in early adolescence.

**Table 1:** Glossary.

High income countries (HICs)	Countries with a gross national income per capita of $12, 536 or more in 2019 (World Bank Atlas method)
Low- and middle-income countries (LMICs)	Countries with a gross national income lower than $12, 536 per capita (Threshold between low and middle income: $1,036)
Global south	LMICs located in Africa, Asia, Oceania, Latin America, and the Caribbean Mahler, 2017, but not necessarily on the Southern hemisphere
Low-resource settings	Settings characterized by relative lower funds than other settings, but not necessarily pertaining to the Global south
Digital media	Digitized content that can be shared through online networks
Social media	Subset of digital media, related to the online platforms that allow people to create and share information with others and establish social networks Kross et al., 2020
Adolescence	Period of life between 10 and 24 Sawyer et al., 2018 or 25 National Academies of Sciences, 2019 years of age between childhood and adulthood –
Early adolescence	Generally understood as the subset of adolescent years between 10 and 14 years of age (WHO), 10–12 years of age (NASEM), characterized by the onset of puberty (Note: Early adolescence might also be used to refer to those adolescents who reach puberty earlier than their peers)
Very young adolescents	Sometimes used as synonymous with early adolescence, can also include pre-adolescents (8–12 years of age), some overlap with “tweens”

The transition into adolescence constitutes a window of opportunity which can set and reset developmental trajectories with a lasting impact (Dahl et al., 2018). Early adolescence is characterized by a set of transitions—from childhood into adolescence, the onset of puberty, and social transformations. Across cultures and countries, these transitions make early adolescence a developmental period of opportunity, but also of increased risk for behavioral, emotional, and mental health problems (Dahl et al., 2018). These vulnerabilities can be amplified if new technologies—and the ways that youth use these technologies—are not optimized to recognize and minimize these risks. Early adolescence is also a key opportunity period for exploration and learning, particularly related to identity development and the navigation of an increasingly complex social world (Dahl et al., 2018), processes that are transformed by social media (Spies Shapiro and Margolin, 2014).

The impact of social media use in very young (or early) adolescents (VYAs), those between 8 and 12 years of age, has been largely neglected by research, despite the fact that VYAs in LMICs undergo profound changes in access to digital media, are a growing demographic for social media platforms, and represent the most inexperienced adolescent users (Pangrazio and Cardozo-Gaibisso, 2020). VYAs in LMICs are often early adopters of technology and parents and caregivers may lack the learning and expertise necessary to provide effective guidance around social media use (Barbosa, 2014; Livingstone and Byrne, 2015).

A developmental science perspective (Giovanelli et al., 2020) can provide insights into how neurobehavioral changes in early adolescence can interact with social and emotional learning challenges—and with the contexts in which youth are developing. These interactions can create specific risks for digital media use as well as specific opportunities for this developmental stage (see Table 2A). When considering the ways these risks and opportunities may be further amplified in LMICs, it is critical to recognize how specifics of economic and sociocultural contexts shape so many aspects of these learning experiences (see Table 2B). Taken together, this perspective highlights the potential to leverage insights into (1) the social and emotional learning experiences that contribute to developmental inflection points that occur during early adolescence; (2) how changes in digital media use during an inflection point can alter specific trajectories related to health, education, social, and economic success, and (3) the specific ways in which family, social, economic and cultural contexts shape different dimensions of early adolescent development (Bornstein, 2017).

**Table 2A:** Examples of how specific aspects of social emotional learning & identity development in early adolescence create risks and/or opportunities.

**SEL relevant neurobehavioral changes in early adolescence**	**Developmental SEL challenges in early adolescence^a^**	**Digital media risks**	**Digital media opportunities**
Increased sensitivity to social evaluative feedback Increased motivational salience of status, prestige, respect, and belonging Increased novelty-seeking, and exploration Pubertal activation of romantic and sexual motivations	• Learning to navigate increasingly complex social environments • Learning to manage more complex peer relationships; more emotionally charged peer relationships, including emerging romantic and sexual interest	• Cybervictimization and digital drama^b^ • Social media features (including publicness, quantifiability and permanence)^c^ intensify social evaluation and exclusion, as well as the drive for social rewards which can include showcasing risky behavior • Social media add a digital dimension to peer influence, including exposure to peers' risky behavior and mechanisms for peer influence	• Social connection^d^ • Online platforms allow for social skills and relationship practice at a time of expanding peer relationships. These can also create opportunities for scaffolding and monitoring (e.g., by teachers and parents). • Written and asynchronous communication might easier social entry points for shy, isolated, or marginalized adolescents • Exposure to social networks and norms beyond the local physical community
Increased proclivity for social exploration and social risk taking	Self/other learning Identity formation	• Exposure to negative role models • Risk of cybervictimization, especially for marginalized youth • Exposure to hate speech and exploitation • Construction and permanence of digital footprint restricts flexibility and temporality in identity development. • Vulnerability to advertisement targeted at adolescents^e^	• Exposure to positive role models • Digital platform for exploration and experimentation and creative expression^f^^,^^g^ • Exposure to positive and social cultural norms not reflected in the immediate geographic community^h^ • Connecting with similarly minded peers might be associated with increased sense of acceptance • Gamified experiences might be conducive to experiences of mastery
Increased desire for independence and autonomy	• Emerging capacities of cognitive control (particularly under conditions of strong emotion) • Navigating risky environments without adult supervision	• Unsupervised and unmediated time online due to limited parental skills and resources might lead to increased exposure to harmful content^i^ • Overly restrictive parenting can curtail online learning opportunities	• Openness to parental mediation (relative to older adolescents)^j^ • Motivation to develop digital skills and literacy can provide openness to school scaffolding and digital citizenship curricula^k^

**Table 2B:** LMICs considerations that apply broadly to digital media use in VYA.

• Digital divides in use, access and appropriation exacerbate existing disparities (e.g., gender, socioeconomics, educational attainment)^l^^,^^m^^,^^n^ • Digital gender divide^o^ • General and digital literacy might be barriers to access^p^, compounded by undersupply of culturally, linguistically, and regionally tailored content • Transition into secondary school might be a point of vulnerability^q^ • Mobile phone use and subsidized data use by Big tech is especially suited for social media consumption (vs. using the computer which facilitates other activities)^r^ • Cultural norms and values (i.e., individualism vs. collectivism, self enhancement vs. self-transcendence) influence and in turn are influenced by digital media use • Exploration can expand beyond the community^s^ • Digital media enable remote acculturation, which might open avenues for exploration, challenge to local norms and intergenerational discrepancies that might also lead to parent-adolescent conflict^t^ • Gender norms might limit girls' exploration and limit mixed-gender socialization^u^. • Lack of intergenerational knowledge, scaffolding and wisdom related to digital media. Compared to HICs, parents are relatively more inexperienced and tend to be overly restrictive^v^. • Adolescents help parents with technology^w^ and act as online information brokers^x^ • Limited online regulation and safety^y^

a *Dynamic physical and brain maturation contribute to changes in learning and cognitive, emotional, and social, and identity development. Coupled with behavioral and contextual changes, early adolescence is a critical time of heightened sensitivity to information related to the self- and other, increasing the drive for social rewards and sensitivity to social evaluation (Dahl et al., 2018)*.

b *(Patchin, 2020)*.

c *(Nesi et al., 2018a,b)*.

d *(Rideout and Fox, 2018)*.

e *(Rummo et al., 2020)*.

f *(Spies Shapiro and Margolin, 2014)*.

g *(Uhls et al., 2017)*.

h *(Ybarra et al., 2015)*.

i *(Cabello-Hutt et al., 2018)*.

j *(Branje, 2018)*.

k *(Pangrazio and Cardozo-Gaibisso, 2020)*.

l *(Benítez Larghi et al., 2015)*.

m *(Plan International, 2018)*.

n *(Pew Research Center, 2019)*.

o *(Plan International, 2018)*.

p *(Pew Research Center, 2019)*.

q *(Eccles and Roeser, 2011)*.

r *(Romanosky and Chetty, 2018)*.

s *(Manago and Pacheco, 2019)*.

t *(Ferguson and Bornstein, 2012; Asemah et al., 2013)*.

u *(Kågesten et al., 2016)*.

v *(Barbosa, 2014; Livingstone and Byrne, 2015; Cabello-Hutt et al., 2018)*.

w *(McKenzie et al., 2019)*.

x *(Yip et al., 2016)*.

y *(Livingstone et al., 2015)*.

## Digital Media, Development, and Learning

Biological and neurobehavioral changes in the transition years of early adolescence, initiate a set of dynamic changes—not only in growth and physical development but also in learning processes that shape cognitive, emotional, social, and identity development. These formative learning experiences are occurring in specific social, family, peer, and cultural contexts—in ways that shape individual experience and development (Dahl et al., 2018). VYAs have natural proclivities toward greater independence, expanding their sense of self, and exploring ways to navigate the complex social world of adolescence. Social media platforms appeal to these proclivities and play an important role in promoting self-expression and providing a source of social support and privacy from adults (Uhls et al., 2017; Gerwin et al., 2018). In the virtual space, online risks are positively associated with online harms (Livingstone and Helsper, 2009), so curtailing access for protection can limit positive exploration that is necessary for learning and development. Supporting VYAs' use of digital media requires striking a balance between encouraging autonomy and assuring protection. Here we outline key early adolescent developmental processes that could inform policies and practices to transform social media into safe and enriching digital spaces for youth.

### Developmental Challenges of Early Adolescence: Learning to Navigate Complex Social Worlds, Forming Identities, and Increasing Autonomy

A key aspect of adolescent development is the social reorientation from family to peers and other influential adults, as adolescents strive for independence (Fitton et al., 2013; Nelson et al., 2016). Due to changes in socio-emotional learning processes (Blakemore and Mills, 2014; Casey, 2015; Shulman et al., 2016), VYAs experience increased sensitivity to their social world, including social evaluation (Somerville, 2013), and peer influence (Albert et al., 2013). In addition, reward and regulatory mechanisms undergo extensive development throughout adolescence (Casey, 2015). As a result adolescents exhibit emerging capacities of cognitive control, that might be particularly challenged under conditions of strong emotion or reward (Casey, 2015; Dahl et al., 2018). Social and digital media have become “salient developmental contexts” (Vannucci and Ohannessian, 2019), that uniquely appeal to, and are challenged by, the heightened importance of social contexts, and increased sensitivity to rewards, and emerging regulatory capacities. These powerful platforms of exploration and autonomy allow adolescents to build their identity and social worlds against an extended backdrop of complex peer relations, comparison, acceptance and audience (Subrahmanyam et al., 2006; Valkenburg and Peter, 2008; Fitton et al., 2013; Gerwin et al., 2018; Vannucci and Ohannessian, 2019; Pangrazio and Cardozo-Gaibisso, 2020).

Developing technical competence in these digital developmental contexts is important for early adolescent self-esteem, pride, and confidence (Fitton et al., 2013). Online platforms serve as a space to escape parental monitoring, to develop problem solving skills, and to master challenging tasks (Uhls et al., 2017), particularly during the global pandemic (Survey Monkey and Common Sense Media, 2020; Magis-Weinberg et al., 2021). Developing mastery, as well as gaining increased skills and competencies are important VYAs tasks, related to identity formation (Field et al., 1997; Conger et al., 2009), that can be specially scaffolded with digital media. With the rapid expansion of digital learning worldwide in the face of the COVID-19 pandemic, this reality has become even more true. Digital platforms leverage learning associated with two key processes that undergo development during early adolescence - reward processing and competition. By *gamifying* rewards and social evaluation, digital platforms motivate learning through the pursuit of competence and mastery. This builds upon the development of real skills and knowledge along with the feeling of success and growing confidence, contributing to affective learning and shaping identity for VYAs (Sailer et al., 2017; Dahl et al., 2018).

### Social Media: Opportunities for Early Adolescent Social Emotional Learning and Identity Development

Early adolescence is an important inflection point around digital media use, with (1) increasing access and autonomy over mobile devices, particularly in HICs (Rideout and Robb, 2019; Young and Tully, 2019; Smahel et al., 2020) and social media (Odgers and Robb, 2020), (2) lack of effective regulations, and (3) lack of supervision and scaffolding, particularly in LMICs. Digital independence allows for unsupervised technology use, risk-taking, and digital autonomy (Ofcom, 2017; Anderson and Jiang, 2018). Before COVID-19 lockdowns, young people in LMICs reported that mobile devices granted more autonomy and allowed for greater mobility since phones also make them feel safer and able to communicate with parents and peers (Girl Effect and Vodafone Foundation, 2018). In LMICs, connecting online can help reduce loneliness and increase well-being (Girl Effect and Vodafone Foundation, 2018). During lockdown, online tools have become increasingly vital as sources of connection for adolescents (Survey Monkey and Common Sense Media, 2020; Magis-Weinberg, 2021).

Digital media affords online opportunities for youth, including access to information and education, connection with friends, expression of identity, entertainment, creative expression, participation, and engagement (Hasebrink et al., 2008; Livingstone et al., 2017b; Uhls et al., 2017). Social media in particular can help adolescents fine-tune social skills, meet social needs and enhance relatedness (Spies Shapiro and Margolin, 2014; Uhls et al., 2017), as predicted by the *stimulation hypothesis* (Bryant et al., 2006; Valkenburg and Peter, 2007). Social media features transform peer and group dynamics by changing the frequency, quality, intensity, and novelty of social interactions (Nesi et al., 2018a,b). Unlike in-person interactions, online communication allows for remote constant social connection which is also customizable (e.g., deciding to block a friend) (Manago, 2014; Nesi et al., 2018a,b). As VYAs develop social skills, they may benefit from the ability to customize online interactions but can also be challenged by increasing demands for availability or the lack of social cues in online settings. Thus, in contrast to older adolescents, VYAs may require additional support and parental mediation as they enter social media spaces.

Relationships are by-and-large strengthened online, with social media being key in maintaining existing friendships and online social networks mirroring and reinforcing offline social networks (Rideout and Fox, 2018). VYAs constantly connect with friends online, handle life events and strengthen interpersonal ties (Fitton et al., 2013; Vannucci and Ohannessian, 2019). Social media also allows for online-only friendships which can offer VYAs critical support, particularly for adolescents who are marginalized offline (Ybarra et al., 2015; Massing-Schaffer et al., 2020). Digital technologies can also offer new opportunities for mental health interventions with young people, particularly the most marginalized, in LMICs (Giovanelli et al., 2020; Rost et al., 2020). Social media has played a preeminent role as a bridge for physical distancing in the pandemic lockdowns around the world, constituting a protective but also a risk factor for adolescent mental health and well-being (Ellis et al., 2020; Magis-Weinberg et al., 2021; Magson et al., 2021).

Several factors influence the impact of social media on VYAs (Spies Shapiro and Margolin, 2014), including (1) patterns of use, (2) gender, and (3) underlying vulnerabilities and strengths, resulting in a complex landscape of associations with well-being. Patterns of use based on time, number and type of social media platform determine whether psychosocial outcomes are positive or negative. For example, for VYAs in HICs, high social media use across a variety of platforms can be a risk factor for distress but can also enhance friendship competence and support (Vannucci and Ohannessian, 2019). Research in HICs suggests gender differences in motivations driving use. Girls are more likely to turn to social media for interaction than boys, engaging in self-disclosure of feelings and problems (Lenhart, 2015a; Rideout and Fox, 2018), while boys use more video games to interact and enhance their social status (Lenhart, 2015b; Patchin, 2020).

Social media enhances relational interactions for adolescents who are already socially engaged, “rich get richer” (social enhancement hypothesis; Peter et al., 2005; Desjarlais and Willoughby, 2010). However, social media can also amplify isolation and other barriers for lonely adolescents (Jackson, 2007) and negatively impact self-esteem (Valkenburg et al., 2006). Further, the *social compensation* hypothesis posits the use of social media to make up for offline introversion or social anxiety, “poor get richer” (Valkenburg et al., 2006). At the same time, the *displacement* hypothesis (Kraut et al., 1998) proposes that social media can displace time spent on health-promoting activities, including exercise, face-to-face interactions, and educational activities. Research should explore how the different hypotheses predicting the impact of social media on adolescent well-being in HICs (social stimulation, enhancement, compensation, displacement) apply to youth in LMICs. For example, in settings with limited extracurricular activities or safe spaces for youth to meet after school, online platforms do not displace other alternatives, and might be *the only* option for adolescents to meet their friends. Furthermore, the benefits of social compensation through social media might not be as strong in settings where there is heightened value for in person communication.

### Digital Media: Risks for Early Adolescent Social Emotional Learning and Identity Development

Digital and social media are also associated with online risks for dangerous or illicit behavior enabled by the anonymity of the online world (World Health Organization, 2011; Uhls et al., 2017; UNICEF, 2017; Patchin and Hinduja, 2020). Most behaviors and risks online appear to mirror offline activities (for a review in HICs see George and Odgers, 2015). However, in HICs it has been shown that digital media also introduces new risks for adolescents, particularly in relation to body image (Choukas-Bradley et al., 2020), sleep (LeBourgeois et al., 2017), cyberbullying (Patchin and Hinduja, 2020), and digital reputation (George and Odgers, 2015). In adolescence, peer interactions contribute to psychopathology onset and maintenance, and influence risk taking (Gardner and Steinberg, 2005). Thus, some negative aspects of peer relationships can be amplified and transformed by social media, including cybervictimization (Fisher et al., 2016), social exclusion and *digital drama* (Anderson and Jiang, 2018), social comparison and feedback seeking (Nesi and Prinstein, 2015), and risky behaviors (Vannucci et al., 2020).

Especially in lower socio-economic backgrounds, negative online experiences can “spill-over” into negative offline interactions and events (George et al., 2020). Further, although social media platforms are officially restricted for under 13 year-olds, many VYAs still access them, without protections appropriate for their age (Young and Tully, 2019; Odgers and Robb, 2020). The VYAs most vulnerable to online risks include girls, youth marginalized by identity, culture, race, ethnicity or economics, and children with disabilities or those with mental health concerns, compounding with their vulnerability to offline risks as well (UNICEF, 2017). Youth growing up in LMICs may experience greater risks associated with social media use (Livingstone et al., 2017a; Banaji et al., 2018)—because of the digital dimension to existing vulnerabilities, and the fact that they typically have less adult support, scaffolding, and monitoring online (Cabello-Hutt et al., 2018). These vulnerabilities are compounded by the insufficiency of services and policies to address child and adolescent mental health needs (Zhou et al., 2020) despite the fact that they are a leading cause of health-related burden for youth worldwide (GBD Causes of Death Collaborators, 2017). In LMICs, cross-sectional studies conducted with older adolescents in Asia and Latin America have found some evidence of a negative association between screen time and unhealthy behaviors (Yan et al., 2017), and problematic media use and poor mental health (Hanprathet et al., 2015; Oberst et al., 2017; Wang et al., 2018), mediated by the fear of missing out and intensity of social media use (Oberst et al., 2017). Adolescents who use more social media experience the most risks but even more opportunities (Marques et al., 2018).

Opportunities and risks associated with digital media vary depending on the developmental stage and sociocultural context of the user (Cabello-Hutt et al., 2018; Smahel et al., 2020). Older adolescents engage in more online risks, regardless of having more digital skills (Livingstone and Helsper, 2009; Sasson, 2014; Cabello-Hutt et al., 2018). But, in addition to risks, older adolescents also enjoy more online opportunities than early adolescents (Livingstone and Helsper, 2009). Thus, early adolescence is a window of opportunity for promoting digital skills that better prepare adolescents for the online world.

## Contextual and Cultural Factors Broadly Influencing VYA Access, Use and Appropriation of Digital Technology

Adolescent digital ecologies are impacted by individual beliefs, abilities and family dynamics, as well as structural factors related to income, technology access and cultural values (Livingstone et al., 2017a; Banaji et al., 2018; Cabello-Hutt et al., 2018; Marques et al., 2018; Smahel et al., 2020; Manago and McKenzie, under review). Socioeconomic status, educational resources and parental availability, expertise and attitudes toward technology, and mediation inform opportunities and risks for youth's lives online (Lemphane and Prinsloo, 2014; UNICEF, 2017; Banaji et al., 2018; Cabello-Hutt et al., 2018).

Both access and effective use are required to make the most of media. Around the world, economic and sociocultural factors influence multiple digital divides in (1) *access*, (2) *use*, and (3) *appropriation* of digital media that affect early adolescents (Benítez Larghi et al., 2015). Digital divides compound other social and structural inequities such as education, poverty, gender, age and geography (UNICEF, 2017; Banaji et al., 2018). Digital divides have been exacerbated by the pandemic lockdowns and school closures. As of November 2020, two thirds of school-aged youth did not have internet connection at home. This lack of access disproportionately affects 87% of youth in LMICs compared to 6% of youth in HICs, limiting education and connection with peers and family during the global COVID-19 pandemic (UNICEF International Telecommunication Union., 2020). Still, increased access without increased skills and literacy can amplify existing inequalities (Toyama, 2011).

Home computer and tablet access is relatively rare in LMICs (Pew Research Center, 2019). Compared to these platforms, the more common mobile access to the internet provides lower levels of functionality and content availability and the ability to seek and find information (Fong, 2009). Mobile devices facilitate access to social media platforms that are optimized for this medium of delivery. In addition, in many LMICs, cell phone plans provide free access to social media platforms, whereas internet services providers regulate cost and accessibility of third-party platforms and other services (Romanosky and Chetty, 2018). Predominant access through mobile devices contributes to a *smartphone* “bedroom culture,” where use is more private and less supervised (Bovill and Livingstone, 2001). In low education, low-income settings, the use of social media is much more prevalent than other technology-based activities (like looking for information, educational opportunities or interacting with governmental agencies) (UNICEF, 2017; Girl Effect and Vodafone Foundation, 2018; Pew Research Center, 2019).

Emergent in childhood, gender norms and attitudes start to crystalize in early adolescence, as a result of pubertal changes, resulting in an expansion of boys' worlds but a contraction of girls' worlds, particularly in LMICs (Chandra-Mouli et al., 2017). While in Europe there are few gender differences in terms of adolescent technology access (Smahel et al., 2020), in LMICs there is a *digital gender divide* where girls disproportionately face barriers to access, use and appropriation (Plan International, 2018). Adolescent boys are 1.5 times more likely to own a mobile phone than girls, who must borrow devices (Girl Effect and Vodafone Foundation, 2018). Sharing friends and relative's phones gives girls conditional and restricted access, recreating conditions of gender specific community surveillance (Manago and Pacheco, 2019). Girls experience increased online risks and lower opportunities that mimic their offline realities, including community scrutiny and harassment or grooming. Female adolescents are worried about strong backlash responses that aim to reinforce traditional social norms (Girl Effect and Vodafone Foundation, 2018).

In early adolescence, the early adoption of new media, social reorientation, and increased sensitivity to context, may enhance opportunities for culture acquisition (Ferguson and Bornstein, 2012; Worthman and Trang, 2018). Social media practices of early adolescents reflect and are influenced by *local* cultural norms, dimensions, and values (Manago and McKenzie, under review). For example, an emerging literature *with adults* has demonstrated how cultural differences in collectivism *vs*. individualism (Hofstede, 1980) impact features of online networks as well as online behavior. Korean young adults, as part of a collectivistic culture, emphasize the relational aspects of social media, including belongingness, more than US young adults. In contrast, individualistic settings are associated with more direct communication and more open self-disclosure (Huang and Park, 2013; Na et al., 2015; Hong and Na, 2018). Thus, social media can reinforce traditional *local* cultural values (Holmes et al., 2015). Digital media also enables *remote* acculturation by permitting indirect intercultural contact across geographic distance (Ferguson and Bornstein, 2012). For instance, early adolescents in Jamaica can develop an orientation toward U.S. culture through mass media, without necessarily traveling abroad (Ferguson and Bornstein, 2012, 2015). As early adolescents are actively learning about themselves and their social worlds, it is important to examine the effects of how the indirect exposure to multiple cultures through digital media informs identity development, relationships with peers and families.

Given the increased motivation to gain social value in adolescence, it is important to recognize the strong socio-cultural influences on the ways to enhance self-evaluation (Becker et al., 2014) in the continuum between openness to change *vs*. conservation and self-enhancement *vs*. self-transcendence (Schwartz, 2012). For example, in a particular cultural context is an adolescent's positive self-evaluation based more on doing her duty or on controlling her life? This could play out in the way young adolescents seek social value enhancement in the digital domain. It could also play out in the opposite way—creating conflicts between the social-value currencies online and those in the local cultures. There are similar issues with gender norms, values, and behaviors (Abiala and Hernwall, 2013). Context and religious influences can amplify some traditional cultural values (Schwartz, 2012), and more specifically the opportunities for gaining social value as an adolescent girl vs. a boy (Kågesten et al., 2016). For example, some cultures value bold behavior in boys, but shy behavior in girls. These can shape the social learning opportunities and vulnerabilities in LMICs and can create or amplify conflicts with the local culture. There is also emerging evidence of the influence of media on gender norms in early adolescence (Kågesten et al., 2016; Livingstone et al., 2017a). Reshaping of relationships with families might introduce intergenerational discrepancies and result in parent-child conflict (Ferguson and Bornstein, 2012; Manago and McKenzie, under review).

## New Directions: Investigating Specific Impacts of Digital and Social Media on Early Adolescents in LMICS

Early adolescence is a time of mental health symptom onset (Paus et al., 2008), with increased frequencies of internalizing and externalizing problems (McLaughlin and King, 2015; Petersen et al., 2015). While the impact of social media on well-being, particularly mental health, has been an important focus of research in HICs, this question is underexplored in LMICs (Odgers and Jensen, 2020; Orben, 2020). In HICs, in work mostly with older adolescents, there is a negative (but small and non-causal) association between social media use and well-being [see recent reviews by Ivie et al. (2020), Odgers and Jensen (2020), Orben (2020), and Schønning et al. (2020)]. However, manifestations of psychopathology might be different for younger vs. older adolescents (American Psychiatric Association, 2013), and hence it is necessary to have a more granular study of the relationship between digital media and well-being for VYAs and consider developmental trajectories of psychopathology. Emerging evidence in the USA suggests that social media use might be associated with externalizing behaviors, including poor behavioral conduct and delinquent behavior in early adolescence (Ohannessian and Vannucci, 2020). There is currently a paucity of work on VYAs in general, and the Global South in particular (see Schønning et al., 2020, where only around 5% of the studies were conducted in early adolescence in the Global South—all of them in Asia).

There is an urgent need for work on early adolescence in a broader set of contexts in the Global South. Given that social media is the predominant digital media use for youth in these settings, understanding its relationship with adolescent well-being is paramount. Future studies should leverage the important recent advancements in research practices (Kross et al., 2020) which include moving away from coarse and subjective measures of screen-time. Studies should incorporate objective measures of media use, focus on longitudinal and experimental designs that are suited to assess causality, and align with principles of transparent and reproducible data analysis (Odgers and Jensen, 2020; Orben, 2020). It is important to incorporate an individual differences approach to identify sources of vulnerability and resilience, beyond average effects. Crucially, studies should focus on diverse and under-represented youth around the world and incorporate a socio-cultural and developmental approach (Manago and McKenzie, under review).

The heterogeneous effects of social media on well-being relate to the different psychological processes at play as users navigate this emerging social ecosystem (Kross et al., 2020). For example, positive online experiences reduce loneliness for early adolescents in Peru, with opposite effects for negative interactions (Magis-Weinberg et al., 2021). Early adolescent use of social media for comparison with others is associated with depression above and beyond a simple measure of screen time (Magis-Weinberg et al., in prep). Research should incorporate more careful consideration of different dimensions of digital media (patterns of use, motivations, positive and negative dimensions, passive vs. active use, etc.) to fully characterize its positive and negative consequences (Kross et al., 2020; Odgers and Jensen, 2020; Orben, 2020). In Tables 2A,B, we highlight several dimensions of VYA development that might inform risks and opportunities and warrant further investigation in LMICs. Future research should consider not only the heterogeneity that exists *between* HIC and LMICs, but also *within* LMICs. More research is needed to further delineate the *general principles* operating through early adolescent developmental processes of socio-emotional and identity learning and well-being in relation to digital media use. More research is also needed that focuses on how these principles apply in *specific* ways within particular contexts, countries, and cultures. Achieving a deeper understanding of these interactions in LMIC settings, can inform innovative approaches to promote early learning and development of adaptive digital skills and discernment.

## Building Skills and Resilience for Constructive Engagement Online in LMICS

Given the persistent global expansion of digital media use, there is an opportunity to leverage developmental science to inform policies and practices that create safe and enriching digital spaces. Enhancing opportunities for scaffolded learning to promote the early development of technology relevant knowledge and skills in VYAs has multiple benefits. This improves individuals' abilities to find and effectively learn, contribute, and connect, while also improving their capacities for avoiding harms. A developmental science approach^2^ is crucial to ensure that the evolving adolescent needs and capacities do not lag behind design and policy considerations for younger children which might be focused on safety and reliant on parental controls. Developing policies and practices that help to provide these opportunities for VYAs can be particularly challenging in many LMIC settings—yet the importance is even greater because of the combination of increased vulnerabilities and opportunities.

Despite reorientation toward peers, in the transition from childhood into adolescence caregivers still play an important role in supporting learning, setting limits, and influencing behavior (Branje, 2018). Early adolescence offers an opportunity for more parental and teacher mediation and guidance during exploration of digital media, making it a prime time for intervention and setting of future trajectories (Odgers and Robb, 2020). It is therefore crucial to build a strong evidence base that can support culturally appropriate recommendations for parental and teacher guidance to promote positive media use, digital citizenship and literacy in LMICs. Special attention should be paid to addressing the gender digital divide in LMICs.

Across the globe, VYAs experience transitions in school to larger, more complex social environments, or to a complete departure from formal education (Eccles and Roeser, 2011; UNESCO Institute for Statistics, 2018). Given the multiple transitions, it becomes important to find ways to support adaptive learning—and there are ways where digital media can contribute. Schools can serve as spaces where early adolescents can learn digital habits and get formed as digital citizens and can offer VYAs important guiding and support from teachers (Cabello-Hutt et al., 2018; Pangrazio and Cardozo-Gaibisso, 2020). In HICs there has been a proliferation of school-curricula to promote digital citizenship, media literacy and online safety, with limited evidence of their efficacy (Bulger and Davison, 2018). This presents an opportunity to tailor and redesign materials to cater to the specific needs of LMICs while building the body of evidence about their impact. Researcher-practitioner collaborations with schools in LMICs can advance design, implementation, and evaluation of school-curricula and programs to foster abilities around balance, online privacy and safety, online relationships and well-being and digital literacy (Magis-Weinberg, 2021). These programs can be more effective if grounded on developmental science principles and tailored to different stages (Pangrazio and Cardozo-Gaibisso, 2020). Crucially, digital technologies present a unique opportunity for learning experiences that go beyond classroom education on digital literacy to provide *experiential* learning (Kolb, 2014) in a simulated but realistic environment. For example, *Social Media TestDrive* (DiFranzo et al., 2019) is an interactive simulation that combines social media and educational components. In this safe environment, young adolescents get hands-on experience and guided reflections on the risks and benefits of social media. Like a driver simulation, this allows for learning to navigate the opportunities and vulnerabilities on the “digital highways,” building effective skills early on (Ribble et al., 2004). This can set early adolescents on positive learning trajectories as they get more experience in social media and online relationships later in adolescence.

## Conclusion

There is a compelling need to better equip young adolescents to successfully navigate the risks and the opportunities of the digital world. This challenge is compounded by the rapid pace of change, variability across global contexts in the ways digital devices are becoming available and used by youth, and limited high-quality global data capturing these dynamic changes in the wave of technological uptake. Focusing on early adolescence as a time of opportunities—a period of rapid growth, learning, and exploration online when youth are developing goals, values, priorities, and are open to adult guidance—creates an exciting approach to early intervention and prevention for digital well-being.

## Data Availability Statement

The original contributions presented in the study are included in the article/supplementary material, further inquiries can be directed to the corresponding author/s.

## Author Contributions

LM-W, ABS, and RD contributed to conception, design of the manuscript, and wrote sections of the manuscript. LM-W wrote the first draft of the manuscript. All authors contributed to manuscript revision, read, and approved the submitted version.

## Conflict of Interest

The authors declare that the research was conducted in the absence of any commercial or financial relationships that could be construed as a potential conflict of interest.

## Footnotes

^1^See Table 1 (Glossary) for definitions and some considerations about key terms.

^2^For a concrete example of policy that incorporates a developmental approach in HICs, see the *Age appropriate design: a code of practice for online services* published in the United Kingdom (Information Commissioners Office, 2020). This regulatory document delineates standards to safeguard minors within the digital world in developmentally appropriate ways by recognizing different needs and abilities in five groups: (1) the pre-literate and early literacy stage (2) core primary school years, (3) the transition years (what we refer to as VYAs), (4) teens, and (5) approaching adulthood (Information Commissioners Office, 2020).
